# Inhibitory Control in Children 4–10 Years of Age: Evidence From Functional Near-Infrared Spectroscopy Task-Based Observations

**DOI:** 10.3389/fnhum.2021.798358

**Published:** 2022-01-03

**Authors:** Xin Zhou, Elizabeth M. Planalp, Lauren Heinrich, Colleen Pletcher, Marissa DiPiero, Andrew L. Alexander, Ruth Y. Litovsky, Douglas C. Dean

**Affiliations:** ^1^Waisman Center, University of Wisconsin–Madison, Madison, WI, United States; ^2^Neuroscience Training Program, University of Wisconsin–Madison, Madison, WI, United States; ^3^Department of Psychiatry, University of Wisconsin School of Medicine and Public Health, Madison, WI, United States; ^4^Department of Medical Physics, University of Wisconsin–Madison, Madison, WI, United States; ^5^Division of Otolaryngology, Department of Surgery, University of Wisconsin–Madison, Madison, WI, United States; ^6^Department of Communication Sciences and Disorders, University of Wisconsin–Madison, Madison, WI, United States; ^7^Division of Neonatology & Newborn Nursery, Department of Pediatrics, University of Wisconsin–Madison, Madison, WI, United States

**Keywords:** executive function, inhibitory control, functional near-infrared spectroscopy, development, multimodal Go/NoGo

## Abstract

Executive function (EF) is essential to child development, with associated skills beginning to emerge in the first few years of life and continuing to develop into adolescence and adulthood. The prefrontal cortex (PFC), which follows a neurodevelopmental timeline similar to EF, plays an important role in the development of EF. However, limited research has examined prefrontal function in young children due to limitations of currently available neuroimaging techniques such as functional resonance magnetic imaging (fMRI). The current study developed and applied a multimodal Go/NoGo task to examine the EF component of inhibitory control in children 4–10 years of age. Cortical activity was measured using a non-invasive and child-friendly neuroimaging technique – functional near-infrared spectroscopy (fNIRS). Children’s response accuracy and reaction times were captured during the fNIRS session and compared with responses obtained using the standardized assessments from NIH Toolbox cognition battery. Results showed significant correlations between the behavioral measures during the fNIRS session and the standardized EF assessments, in line with our expectations. Results from fNIRS measures demonstrated a significant, age-independent effect of inhibitory control (IC) in the right PFC (rPFC), and an age-dependent effect in the left orbitofrontal cortex (lOFC), consistent with results in previous studies using fNIRS and fMRI. Thus, the new task designed for fNIRS was suitable for examining IC in young children, and results showed that fNIRS measures can reveal prefrontal IC function.

## Introduction

Executive function (EF) refers to a family of top-down cognitive processes necessary for the control and regulation of behavior and goal-directed tasks (see reviews by Diamond, 2013). EF is often delineated into three core components: (1) inhibitory control (IC), (2) updating or monitoring working memory, and (3) cognitive flexibility between mental sets (Alvarez and Emory, 2006). EF emerges in the first few years of life and continues to strengthen throughout childhood and adolescence (see reviews by Best and Miller, 2010). The development of EF in children is essential as these skills play an important role in multiple cognitive domains and have been associated with long-term outcomes, including school readiness and academic performance (see reviews by Blair, 2016) and overall mental and physical health (Johnson, 2012; Allan et al., 2016). Human behaviors are strongly rooted in the brain; thus, the emergence of EF during childhood is likely closely tied to the development of underlying brain structures and neural networks, particularly in the prefrontal cortex (PFC; Miller and Cohen, 2001; Diamond, 2002; Gogtay et al., 2004). However, it has been challenging to assess neural correlates of EF in younger children, especially preschoolers, partially due to constraints of imaging technology and the lack of age-appropriate tasks that are also easy to conduct in neuroimaging sessions. This study aims to develop a protocol to specifically examine a core EF component – IC in typically developing children 4–10 years of age, using functional near-infrared spectroscopy (fNIRS), which is a non-invasive and child-friendly neuroimaging technique.

Inhibitory control refers to the ability to actively suppress or delay one’s response in order to achieve a goal. IC follows a prolonged developmental trajectory, emerging near the end of the first year before undergoing rapid maturation during the toddler and preschool years, and continuing into adolescence and early adulthood (Kochanska et al., 1996; Williams et al., 1999; Whedon et al., 2020). In particular, two types of IC have been proposed – *response inhibition*, or the ability to stop impulses and prepotent responses, *and interference suppression*, the ability to resist interference from misleading or irrelevant information. At the behavioral level, r*esponse inhibition* has often been examined using a Go/NoGo task, for which participants are required to respond to certain stimuli but not to others, or a stop-signal task when responses are initiated but have to pause for a stop signal (Logan et al., 1997). *Interference suppression*, on the other hand, can be distinguished using a Flanker test (Eriksen and Eriksen, 1974), which assesses resistance to distractor interference by using an array of letters, arrows, or shapes. Multiple image objects are presented, with the center object being congruent or incongruent with the rest. Participants are required to respond to one object by location and ignore the distraction from the rest. A color/word Stroop test has also been implemented to tap *interference suppression* (Stroop, 1935), during which color words are printed in incongruent ink colors. For instance, the word ‘red’ is printed in green ink; participants are required to read the ink color but not the word, with the latter being an automatic process. As the processes of color-naming and word-reading are separate, when they are incongruent, the processing of one will impede the processing of the other and requires enough cognitive flexibility to succeed. For both Flanker and Stroop tests, worse IC is reflected by observing interference operationalized as longer behavioral reaction times (RTs) and/or poorer response accuracies in the incongruent condition compared to the congruent or neutral conditions.

Advances in EF coincide with the rapid structural neurodevelopment of the PFC (Gogtay et al., 2004), with evidence from multi-modal neuroimaging studies supporting the involvement of the PFC in IC tasks (see reviews by Miller and Cohen, 2001; Munakata et al., 2011). *Response inhibition* is associated with the functions in the right PFC (Aron et al., 2004, 2014) and right frontal-striatal regions (Garavan et al., 1999; Durston et al., 2002). Specifically, patients with damage in the right PFC had impaired performance in the Go/NoGo or stop-signal tasks, i.e., failed IC (see reviews by Dillon and Pizzagalli, 2007). Whereas studies using transcranial direct current stimulation (tDCS) to elevate or suppress the local neural activity in the left dorsolateral PFC (DLPFC) in individuals with attention deficit/hyperactivity disorder (ADHD) have shown improved or worsened performance in response inhibition (Soltaninejad et al., 2019; Nejati et al., 2020). These results suggest that the left and right DLPFC may have different roles in cognitive control.

Functional magnetic resonance imaging (fMRI) studies suggest that *interference suppression* is evident in the anterior cingulate cortex (ACC), such that there were greater responses in the ACC during incongruent versus congruent conditions (MacDonald et al., 2000). Another fMRI study found that the differences in activity in the left ACC between color-naming and word-reading increased with age for children and young adults between 7 and 22 years of age (Adleman et al., 2002), suggesting developmental trends for neural underpinnings of EF from early childhood. In the same study, MacDonald et al. (2000) found greater responses in the left DLPFC in the color-naming versus word-reading, suggesting the effect of control of prepotent responses. Also using tDCS (Loftus et al., 2015) or repetitive transcranial magnetic stimulation (rTMS; Vanderhasselt et al., 2006) to directly stimulate the left DLPFC of healthy adults, found improved reaction times but no different Stroop interference effect. Mansouri et al. (2009) summarized previous research and proposed that IC was differentially associated with activity in the left and right, such that the left DLPFC may be associated with the expectation and preparation for upcoming changes in the attentional set (see reviews by Vanderhasselt et al., 2009), i.e., conflict monitoring, whereas the right DLPFC was associated with top-down control and regulation of behavioral responses.

There are challenges in understanding the interactional nature of IC and neural development in children, largely due to measurement issues. For example, the color/word Stroop task is suitable for children above 7 years of age who can read but not younger children (Comalli et al., 1962; Adleman et al., 2002; Homack and Riccio, 2004). Therefore, a day/night Stroop task (Gerstadt et al., 1994) was developed to examine IC in children between 3 and 7 years of age (see reviews by Montgomery and Koeltzow, 2010).

Further, neuroimaging with infants and young children using fMRI has been extremely challenging, as fMRI requires participants to remain very still in the scanner. Thus, fMRI in infants and preschoolers has been restricted mainly to studies during sleep (see reviews by Graham et al., 2015), or in awake infants while passively perceiving stimuli (Deen et al., 2017; Ellis et al., 2020), rather than while completing a task inside the scanner. To date, the majority of neuroimaging studies examining the neurodevelopment of IC processes in preschoolers have been performed using electroencephalography (EEG) based methods (Morasch and Bell, 2011; Watson and Bell, 2013; Liu et al., 2015). These studies demonstrated that the PFC was involved for IC in preschoolers and that variances in EEG measures of verbal and non-verbal IC were predictive of variances in behaviors. These studies have been important for elucidating the brain’s involvement in inhibitory processes; however, the limited spatial resolution of EEG has made it difficult to identify anatomical regions underlying these abilities. Therefore, questions regarding the neural bases of these processes, and their development, remain.

We aim to overcome the aforementioned limitations in understanding the neurodevelopment of IC in children by using a child-friendly, task-based, fNIRS neuroimaging approach. fNIRS is a non-invasive imaging method that utilizes near-infrared light to indirectly measure concentration changes of oxygenated and deoxygenated hemoglobin, denoted as ΔHbO and ΔHbR, respectively, in the local cortical area contained within the pathway of the infrared light. The concentration changes in ΔHbO and ΔHbR are thought to be closely related to the neuronal activity in the cerebral tissue through neural vascular coupling (Wolf et al., 2002). Compared to fMRI, fNIRS is quiet and less sensitive to movement, thus allowing testing of children while awake and performing tasks in a friendly non-isolating environment. In the last decade, fNIRS has been implemented to examine EF in children (Inoue et al., 2012; Anderson et al., 2014; Perlman et al., 2014, 2015; Li et al., 2017; Fishburn et al., 2019). For instance, Li et al. (2017) used a Pet Stroop task to examine EF in 3- to 5-year-old preschoolers. In that study, one of four animal images (cat, dog, frog or bird) were presented from the center of a computer monitor, while four cages each with one animal image were positioned at one of the four corners. During testing, the animal at the center made a sound from its own species or the sound from another species, e.g., a cat said a ‘meow’ or ‘woof’ sound, hence congruent or incongruent trial types. The task was to position the icon of the center animal image back to one of the cages at the corner according to the sound they made (and ignore the prepotent images in the incongruent condition). Their results found greater fNIRS responses in the incongruent versus congruent condition in one channel on the left DLPFC. However, as the task in Li et al. (2017) involved a forced-choice task with four options, hence quite complicated for children at young ages who had poor EF. Indeed, a group of 46 child participants in that study had only mean scores of 74.2% and 67% correct in the congruent and incongruent conditions, respectively. In another study, Fishburn et al. (2019) examined cortical activity in the PFC during IC using a Go/NoGo task. Children were required to respond to images of sunshine (Go) and to avoid responding for images of occasional rainclouds (NoGo). Their results showed that fNIRS measures of IC in the PFC were correlated with parents’ reported anger/frustration control of children. Thus, to date, there has not been a study using fNIRS to directly compare children’s behavioral performance while performing a customized task with standardized assessments of EF especially IC.

In the current study, we developed a neuroimaging protocol using fNIRS to examine EF in children 4–10 years of age. Specifically, a multimodal Go/NoGo task was designed, by combining a cat/dog image-sound-location Stroop test with a Go/NoGo response test. The location of cat/dog image on the monitor in the visual modality and the location of loudspeaker through which meow/bark sound was played in the auditory modality provide spatial cues of the objects. Multimodal components, i.e., sounds, images and locations, were included to engage children during testing to avoid fatigue and boredom. The Go/NoGo component was adopted to simplify responses for children especially at younger ages and to avoid articulation that was involved in the traditional Stroop tasks.

Two goals were addressed: (1) we aimed to validate that the designed task could reveal the development of IC that was consistent with standardized behavioral measures using the NIH Toolbox cognition battery in this group of children, and (2) we wish to establish fNIRS as a valid tool to examine IC by comparing our results with results from previous research and the brain functions related to EF which have been proposed for the PFC. To validate measures from this ‘novel’ task, we first examined the standardized assessments from NIH Toolbox cognition battery, with the expectation that all assessments would reveal similar and positive relations between EF and child age. We then compared the behavioral performance of children recorded in the designed tasks with the standardized assessments and expected that both tests were able to reveal EF in children. To achieve our second goal, we tested the hypothesis that successful recognition of the interferences and IC were associated with maturation of the PFC in children, manifested as longer behavioral RTs and greater activity in the PFC in the incongruent versus congruent condition. Further, we hypothesized that the recognition and processing of interference is also related to the maturation of EF in children, and that older children who are more matured in the PFC compared to younger children could recognize the inferences quicker in the designed task. Therefore, we predicted they would show different response patterns in the PFC as measured using fNIRS.

## Materials and Methods

### Participants

The study included 32 children (14 females) 4.0–10.8 years of age (mean = 6.8 years, SD = 1.9 years). Children were recruited from Waisman Center recruitment registries and via mass email distributed to university employees and students. Twenty-three children (71.88%) were white, two (6.25%) were Asians, and seven children (21.88%) did not report their race. The mothers of all children had an average of 22 ± 1.25 (range: 20–24) years of education (postgraduate level). On the same day of testing for this study, children also participated in another project at Waisman Center involving MRI scanning. Inclusion criteria included being safe to undergo MRI scanning and never having an abnormal MRI before, physically healthy, typically developing, and speaking English as their first language. Exclusion criteria included having been diagnosed with a psychiatric or neurological illness; developmental disorder; having had brain or cardiac surgeries; or mothers reporting having medical conditions, dangerous infections or significant illness during pregnancy. One child (male, 5 years old) failed to complete the task and his data was excluded. There was no difference in the mean ages between females (*n* = 14) and males (*n* = 17) with data that were included [*t*(29) = 1.33, *p* = 0.19]. Experimental protocols were within standards set by the National Institutes of Health and approved by the University of Wisconsin–Madison’s Human Subjects Institutional Review board. The parent or primary caregiver provided written consent for each child participant.

### NIH Toolbox Cognition Battery

Children completed NIH Toolbox assessments (Gershon et al., 2013) using an iPad Pro (11 inches) in a quiet playroom. A trained researcher led the children through the NIH Toolbox instructions for each test step by step until children could understand and felt comfortable performing the tasks. The tasks they completed included a Dimensional Change card sort test that assesses cognitive flexibility (Frye et al., 1995; Zelazo, 2006), a Flanker inhibitory test that assesses EF and IC (Eriksen and Eriksen, 1974), and a List Sorting working memory test that assesses information processing and storage (Tulsky et al., 2013). For the details about each test, please see Zelazo et al. (2013). One child did not complete the Flanker inhibitory test, and two children did not complete the List Sorting test due to App interruption from the iPad. All tests provide raw scores, uncorrected standard scores, and age-corrected standard scores (Casaletto et al., 2015).

### Functional Near-Infrared Spectroscopy Measures of Cortical Activity

#### Multimodal Go/NoGo Task

**FIGURE 1:**
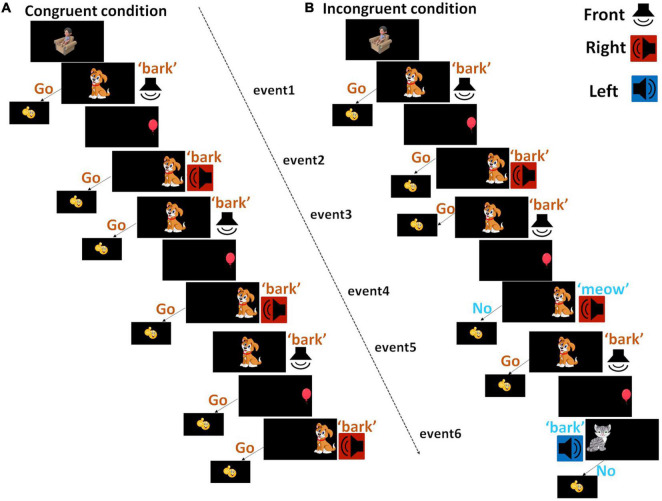
Diagram of testing conditions. The image of a cartoon child sitting in the armchair before the onset of each trial was aimed to remind children to sit still during fNIRS data collection. Images were presented at the center, left, or right side of the monitor. Sounds were presented from loudspeakers at the front (white), left (blue) or right (red) side. **(A)** A congruent trial started with a dog appearing at the center of the monitor, with a bark sound being played from a front loudspeaker. Then a balloon was shown on the left or right side of the monitor, followed by the dog image appearing on the same side and a loudspeaker playing sound from the corresponding location. Different from the congruent trial, **(B)** in an incongruent trial (blue), after the balloon, children might see a dog and hear a meow, or see a cat and hear a bark, at the same or different locations as the balloon. A congruent block consisted of three congruent trials; an incongruent trial consisted of one congruent trial and two incongruent trials. Each trial consisted of two events that required response, i.e., Go for the congruent and NoGo for the incongruent events, from children.

Children completed the Go/NoGo task in a sound-treated booth. For this task, images were presented at the center, left, or right side of a computer monitor in front of the child at 1.5 m distance (Figure 1). Sounds were presented at a comfortably loud level from a loudspeaker (Tannoy Reveal 402) positioned either in front (0° azimuth), on the left (320° azimuth) or on the right (40° azimuth). The positions of images on the monitor and the positions of loudspeakers provide spatial cues of the objects. A congruent trial included two events that required responses and lasted for 6.9 s, as shown in Figure 1. First, a dog image was shown at the center of the computer monitor, followed by a bark sound played from the front loudspeaker, with a 0.35 s delay, then the dog disappeared over a duration of 2.35 s. Children were trained to respond by pushing a one-button computer mouse when seeing a dog and hearing a bark – ‘Go,’ within 2 s after the onset of the sound and before the disappearance of the dog. Then a red balloon appeared on the left or right side of the monitor for 0.6 s, which was used as an informative cue to indicate the location for the forthcoming objects. Immediately after that, the dog image as above was shown where the balloon was, followed by a bark sound played from the loudspeaker at the corresponding location, i.e., congruent sound and image of the same object, with congruent visual and auditory locations.

Two different types of incongruent trials were implemented. For the first type, after the balloon, children might see a dog and hear a meow sound, or see a cat and hear a bark sound, i.e., incongruent images and sounds. For the second type, besides the mismatch of the sound and image, the informative cue about the location was half of the time incorrect (on the opposite side). Children were trained not to respond to incongruent sounds and images – ‘NoGo.’ Though correct responses were only judged based on sounds and images, we introduced extra interference between informative cues and forthcoming events – interferences in spatial locations for two reasons. We aimed to design a task engaging enough for children at both younger and older ages. At the same time, we wanted the task easy to complete, therefore even children did not recognize the conflict in the location cues that are relatively harder compared to images and sounds, they could still perform the task. For simplicity, we will use ‘Go’ events to refer to congruent sounds and images, and ‘NoGo’ events to refer to incongruent sounds and images hereafter. When children responded correctly, i.e., responded to a ‘Go’ event and not responded to a ‘NoGo’ event, a smiley face (emoji) would appear on the monitor for 0.6 s. Otherwise, nothing would be shown on the monitor.

A block design was used for fNIRS data collection. Eight blocks of data were collected per condition across two 5-min sessions, with four blocks per condition presented in a random order in a session. A congruent block (20.7 s) consists of three congruent trials hence six ‘Go’ events (Figure 1). An incongruent block started with a congruent trial, followed by two different incongruent trials hence a total of four ‘Go’ events and two ‘NoGo’ events. The order of the two types of incongruent trials was randomized across blocks. In the same block, the same dog image was presented. Across blocks, four different dog images and four different cat images were used to minimize boredom effects. After the offset of each block, there was a 12-s baseline when children were seeing four different and irreverent cartoon pictures. To add to the developmental appropriateness of the task, we included a cartoon image with a child sitting in an armchair before the onset of each block to remind children to remain sitting well during the task.

Before data collection, children learned the rules of the task at their own pace in the same playroom where they completed the NIH standardized assessments. Once the experimenter was confident that the child was familiar with the rules, the child was taken to the fNIRS booth for another practice session. The practice session consisted of three congruent blocks and two incongruent blocks and ran at the same pace as the real testing, except that no fNIRS data was recorded. Throughout the learning, practicing, and testing phases, the ratio of congruent to incongruent trials was approximately 2.5:1. The reason for having more congruent trials was to increase the sensitivity to goal maintenance and to further build up stronger contrast of conflicts when the forthcoming events are unanticipated or incongruent (Kane and Engle, 2003).

#### Functional Near-Infrared Spectroscopy Data Acquisition

**FIGURE 2:**
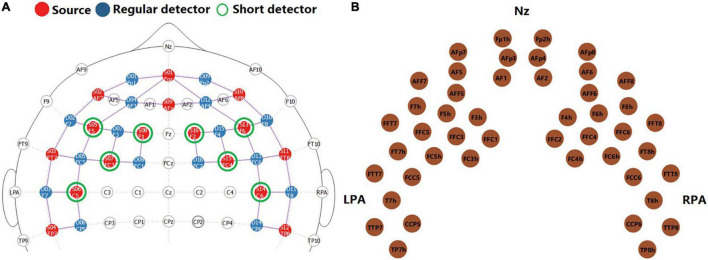
Functional near-infrared spectroscopy montage. Panel **(A)** shows the locations of light sources (red, *n* = 16), regular APD detectors (blue, *n* = 16), and short-channel detectors (green, *n* = 8) on the 10–10 system. This figure was adapted from NIRSite software (NIRSite 2.0, NIRx Medical Technologies, LLC). Panel **(B)** shows the middle point of each pair of light source and detector at 3 cm distance, i.e., fNIRS channels (*n* = 46).

Data were collected in a sound-treated booth, using a continuous-wave NIRScout system (NIRx, Medical Technologies, LLC). Sixteen light sources that emitted near-infrared light of two different wavelengths (760 nm and 850 nm) and 16 avalanche photodiode (APD) detectors were arranged to cover the frontal and temporal cortex on both hemispheres. The locations of light sources and detectors on the 10–10 system are shown in Figure 2A. Each pair of a light source and an adjacent APD detector at around 3 cm distance provided one fNIRS channel that collected signals. The middle points of fNIRS channels are shown in Figure 2B. An extra detector was split into 8 detectors, each of which was connected to one light source at an 8-mm distance, providing a short channel aiming to monitor responses in the extracerebral tissue (Goodwin et al., 2014; Brigadoi and Cooper, 2015). The same short channels were used for data collection and signal processing in Zhou et al. (2020) and have been shown to greatly improve fNIRS signal quality. The optodes were held by an elastic NIRScap of pre-determined sizes based on each child’s head circumference. Before data collection, the light intensity in each channel was checked. For children with dense hair that could affect light intensity, hairpins were used to hold hair to the back of the head where there were no optodes. For channels with poor intensity, the cap was readjusted with the consent of the child.

### Data Analyses

#### Assessments From NIH Toolbox

For the NIH Toolbox cognition battery, the uncorrected scale scores, which were combinations of response accuracies and RTs and assessed the general performance of test-takers, were calculated. Further, the age-corrected scale scores, which compared the score of the test-taker to those in the NIH Toolbox nationwide representatives within the same age band, were calculated. The national mean and SD of the age-corrected scores were 100 and 15, respectively. Age-corrected scale scores between 85 and 115 were within the average range of performance for each age band.

#### Behavioral Measures From Functional Near-Infrared Spectroscopy Session

The accuracies of responses by pushing the button for a ‘Go’ event and not responding to a ‘NoGo’ event were calculated for each individual. In total, the accuracy results in individuals in each condition were derived from 48 responses (or no responses). Besides the accuracy results, d-prime values, which is a standardized measure of sensitivity in a detection task, were also calculated for individuals as differences in *z*-scores for false alarms and true hits. d-Prime values in individuals were derived from 32 responses to events #4 and #6 for both the congruent and incongruent conditions across eight blocks. In an incongruent block, these two events were two ‘NoGo’ events.

The RTs were calculated for trials when children correctly responded to all ‘Go’ events within the 2-s window. RTs for six ‘Go’ events across eight blocks (*n* = 48) in the congruent condition and for four ‘Go’ events (events #1–3, and #5) across eight blocks in the incongruent condition (*n* = 32) were calculated, thus, a maximum of 80 RTs per child. First, the grand mean and SD of RTs for individuals were calculated and any RTs greater or lower than the mean by 2.5*SD were considered outliers. After excluding the outliers, the mean and standard error of the mean (SEM) of RTs were recalculated. The SEM of RTs were included for further analysis, as variances in RTs have been associated with attention, especially in population with attention deficits (see meta-analyses by Kofler et al., 2013), with smaller variances in RT indicating more stable response hence likely better attention to the task. Differences in response accuracies and RTs from the fNIRS session comparing congruent and incongruent conditions were also examined, though they were not used to tap any components of EF.

To examine the effect of interferences (suppression) on behavioral measures, the mean RT values for event #5 were also calculated, which had a preceding ‘Go’ and ‘NoGo’ event (event #4) in the congruent and incongruent conditions, respectively. The normalized differences in RT values were calculated for individuals, which were the differences in the mean RTs between two conditions divided by the grand mean RTs across two conditions and noted as (InCg^4^Cong^5^ – Cong^4^Cong^5^) RT. Increased RTs following ‘NoGo’ versus ‘Go’ events, i.e., greater than zero (InCg^4^Cong^5^ – Cong^4^Cong^5^) have been proposed to reflect the effect of interference (Pires et al., 2018).

#### Functional Near-Infrared Spectroscopy Data Analysis

The fNIRS signals recorded by the NIRScout system were imported into MATLAB, with scripts written by the authors to pre-process data and exclude channels of poor data quality, and scripts from Homer2 software (Huppert et al., 2009) for computing ΔHbO and ΔHbR. To reduce the systemic responses in the extracerebral tissue in the fNIRS data, a short-channel subtraction method was performed using a principal component analysis (PCA) method on short channels using scripts written by the authors. The same data analysis methods were described in detail in Zhou et al. (2020). The ΔHbO and ΔHbR responses across eight blocks, which consisted of 3-s baseline, 20.9-s stimulation, and 12-s silence, in both the congruent and incongruent conditions, were averaged with 3-s baseline responses being subtracted from each trial. For the blocks that children incorrectly responded to three or more events out of six, ΔHbO and ΔHbR responses were excluded for block-averaging. Among 31 children who completed the fNIRS session, 2 (out of 8) blocks were excluded for five children and four blocks were excluded for another child for the incongruent condition, with no blocks being excluded in the congruent condition.

Each block was divided into three periods. In the first period, within 0–11 s after stimulus onset, events were congruent in both conditions, i.e., pre-incongruence. In the second period, within 11–21 s after stimulus onset, there were two ‘NoGo’ events (events #4 and #6) in the incongruent condition, i.e., intra-incongruence. The third period was after stimulus offset (within 21–30 s after stimulus onset), i.e., post-incongruence. Within each period, the maximum absolute difference in the ΔHbO amplitudes between the incongruent and congruent conditions, i.e., (InCg– Cong) ΔHbO was identified. The current study reported results for ΔHbO but not ΔHbR responses as the two highly correlated both with each other and with measures from fMRI (Huppert et al., 2006; Cui et al., 2011). However, ΔHbR responses had smaller changes in amplitudes (Wolf et al., 2002) and lower signal-to-noise ratios reported in some studies (e.g., Strangman et al., 2002).

#### Statistical Analyses

**FIGURE 3:**
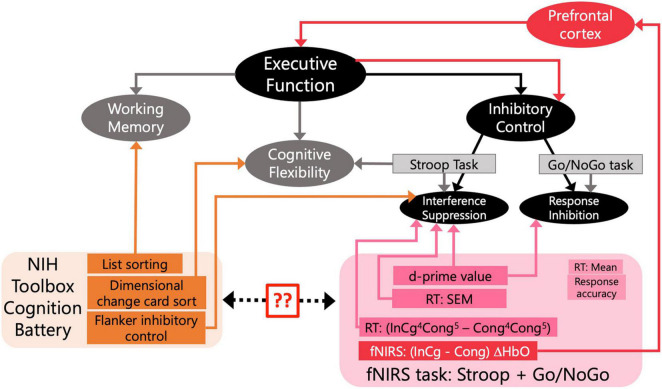
Assessments of executive function (EF) involved in the current study. Rectangles represent assessments that were included to tap constructs of interest. The black and *gray* boxes reflect a conceptual model for EF and standard assessments for inhibitory control (IC). The orange rectangles are the behavioral assessments from the NIH Toolbox included in our study. The pink rectangles are measures from the designed fNIRS task. The two question marks note our first goal to validate the designed fNIRS task by comparing the behavioral measures in the fNIRS session with the standardized assessments from NIH Toolbox. The red lines and arrows note our second goal to tie fNIRS measures (red rectangle) in the PFC (red box) to EF and IC. For measures from our designed task, (InCg^4^Cong^5^-Cong^4^Cong^5^) RT is the normalized difference in reaction times (RTs) between following ‘NoGo’ and ‘Go’ events; RT (SEM) is the standard error of mean for overall RTs; and d-prime is the sensitivity in distinguishing ‘NoGo’ from ‘Go’ events. The mean RT and response accuracy, though included here, were not used to reflect constructs measured as part of EF. For fNIRS responses (red rectangle), differences in the ΔHbO amplitudes between the incongruent and congruent conditions were of interest to examine EF.

Our first goal was to validate the Go/NoGo task we designed to be used during the fNIRS session with three standardized assessments from the NIH Toolbox cognition battery that tap different components of EF (Figure 3). First, we calculated Spearman correlations between the uncorrected scores from the NIH Toolbox cognition battery and the ages of children, the relations between which have been well established (Figure 3). Non-parametric tests were conducted as these measures were non-normally distributed. Second, for behavioral measures recorded from the fNIRS session, we focused on SEM of the overall RTs and d-prime values. Spearman correlations were calculated on the d-prime values and SEM of RTs and the uncorrected scores from three tests in the NIH Toolbox cognition battery, to directly compare the behavioral measures from the designed task with standardized assessments. Holm–Bonferroni method was used to adjust *p*-values for multiple comparisons. We also examined (InCg^4^Cong^5^ – Cong^4^Cong^5^) RTs by conducting a Wilcoxon rank-sum test, with greater than zero (InCg^4^Cong^5^ – Cong^4^Cong^5^) RTs suggesting the effect of IC. To further examine the effect of age differences, we calculated the Spearman correlation between child age and the normalized (InCg^4^Cong^5^ – Cong^4^Cong^5^) RT in the designed task.

To address our second goal and examine fNIRS measures of IC in the PFC (Figure 3), Wilcoxon signed-rank tests were conducted on the (InCg – Cong) ΔHbO amplitudes between the post-incongruence and pre-incongruence periods for each channel. Greater post-incongruence (InCg – Cong) ΔHbO amplitude would be due to IC in the incongruent versus congruent conditions, compared to that in the pre-incongruence period. Holm–Bonferroni method was used to adjust the *p*-values for multiple comparisons. We further examined the correlations between the behavioral measures from the fNIRS session (i.e., d-prime values and SEM of RTs) and post-incongruence (InCg – Cong) ΔHbO amplitudes in brain regions that showed significant effect of IC.

To explore the effect of age differences, we calculated the (InCg – Cong) ΔHbO amplitudes in the intra-incongruence period for each channel, with the expectation that older children would be able to recognize the conflicts and interference earlier than children at younger ages. Adjacent channels where significant correlations with age were identified were clustered into a region to reduce family-wise error rates.

## Results

### Results From NIH Toolbox Cognition Battery

**FIGURE 4:**
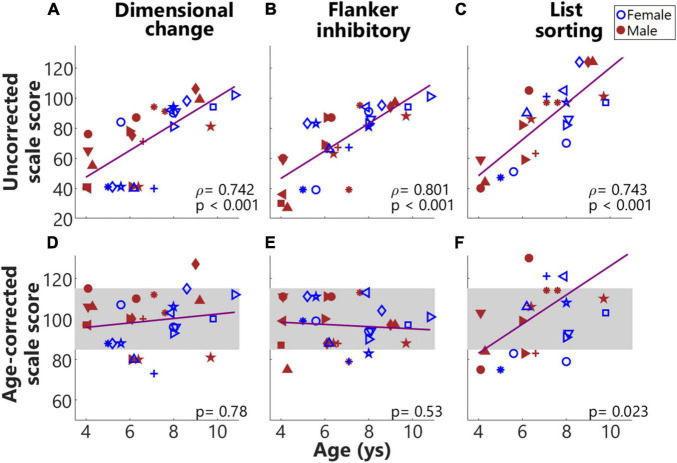
Results from NIH toolbox. **(A–F)** From left to right, each column plots results from the Dimensional change card sort test, Flanker inhibitory attention and control task, and List sorting working memory test. The top and bottom rows show uncorrected and age-corrected scale scores, respectively. Across panels, the same symbols with colors (blue for females and red for males) were used to indicate results from the same individuals. The *gray* rectangles indicate the national average (100) ± 1 SD (15), i.e., 85–115 of the normed scores for the four tasks.

Figure 4 shows the results from NIH Toolbox cognition battery across individuals. As shown in Figure 4, the uncorrected scale scores for all four tasks were significantly and positively correlated (*p* adjusted) with the ages of children, suggesting that as the ages of children increased, they performed better in these tasks. The second row shows the age-corrected scale scores. As shown in Figures 4D–F, the performance of most of the children in these two tasks was within (or close to) the national average range (mean ± SD, the gray rectangle). Table 1 summarizes the results for both the uncorrected and age-corrected scores for each of the NIH Toolbox assessments.

**TABLE 1:** Summary results from the NIH assessments and behavioral measures from fNIRS session.

Assessments		Uncorrected scores		Age-corrected scores	
		Range	Mean ± SD	Range	Mean ± SD
NIH assessments	Dimensional change card sort test	40 – 106	72.33 ± 23.58	73 – 127	98.93 ± 12.61
	Flanker inhibitory attention and control	27 – 101	72.23 ± 22.40	75 – 113	96.87 ± 11.25
	List sorting working memory	40 – 124	84.63 ± 25.42	75 – 146	104.88 ± 21.84
Behavioral measures from fNIRS session	Mean RTs in congruent condition (s)	0.52 – 1.44	0.91 ± 0.24	N/A	
	Mean RTs in incongruent condition	0.55 – 1.60	0.95 ± 0.27		
	(InCg^4^Cong^5^-Cong^4^Cong^5^) RT	−0.22 – 0.62	0.05 ± 0.18		
	SEM of RTs (ms)	27 – 157	77 ± 34		
	d-Prime values	0.31 – 4.31	3.00 ± 1.15		

### Behavioral Results From Functional Near-Infrared Spectroscopy Session

**FIGURE 5:**
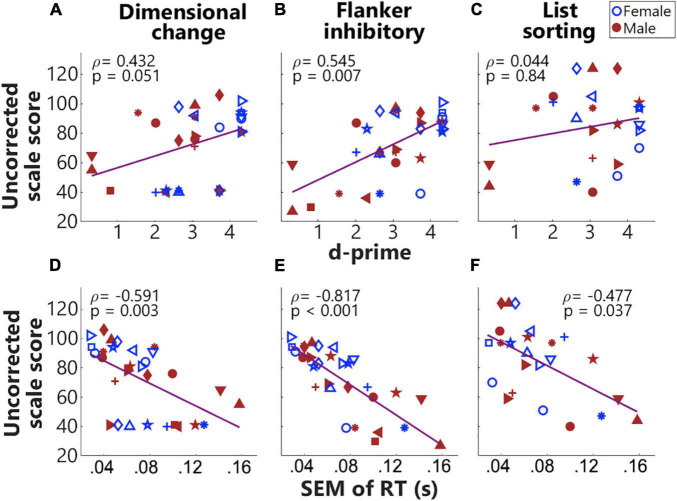
Correlations between behavioral measures in the fNIRS session and standardized assessments in the NIH Toolbox cognition battery. Panels **(A–C)** plot the Spearman correlations between d-prime values and uncorrected scores from the Dimensional change card sort, Flanker inhibitory, and List sorting working memory, respectively. Panels **(D–F)** plot the Spearman correlations for the standard error of mean (SEM) of reaction times (RT)s. The same symbols and colors (blue for females and red for males) were used for individuals as in Figure 1.

Figures 5A–C show Spearman correlations between d-prime values and the three different uncorrected scores from the NIH Toolbox cognition battery (Figure 5). d-Prime values were derived from two ‘Go’ events in the congruent condition and two ‘NoGo’ events in the incongruent conditions. Results showed a significantly positive correlation (*p* adjusted) between d-prime values and the uncorrected scores from the Flanker inhibitory test, with a marginally non-significant correlation with the uncorrected scores from the Dimensional change card sort test. The Dimensional change, and Flanker inhibitory tests tap cognitive flexibility and IC, respectively. As shown in Figures 5D–F, the SEM of RTs were significantly and negatively correlated with all three uncorrected scores. Smaller variances in RT that possibly indicated sustained engagement hence stable responses, predicted better performance in NIH Toolbox cognition tests. As the List sorting test taps working memory, the result that smaller variances in RTs predicted better working memory was not surprising. These significant correlations validated success in the design of this task. Interestingly, three boys (in red) showed low (below-one) d-prime values and large SEM of RTs. These three individuals were around 4 years of age and consistently had floor performance in Dimensional change, Flanker inhibitory (Figure 4), and our designed task (Figure 5), possibly too young to perform these tasks.

**FIGURE 6:**
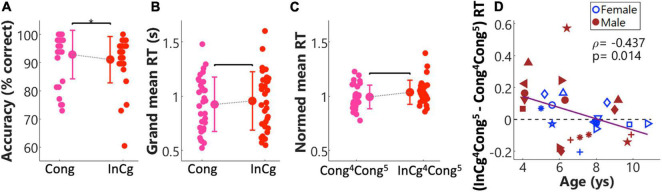
Behavioral results from the fNIRS session. Panels **(A–C)** show the response accuracies, grand reaction times (RTs) across all events and RTs for event 5, respectively, in the congruent (pink) and incongruent (red) conditions. Each panel shows the correlation between the ages of children and their performance. The group means (markers) and standard deviations (SD, bars) for each condition are shown in each panel. Panel **(D)** shows the correlation between the ages of children, and normalized differences in RT for event 5 between the incongruent and congruent conditions, i.e., (InCg^4^Cong^5^ – Cong^4^Cong^5^) RT. For individuals, the symbols used here were the same as in Figure 1.

The response accuracies and mean RTs from the fNIRS session are shown in Figure 6. Figure 6A shows the overall response accuracies for individuals, and the group means and SDs (bars) in the congruent (pink) and incongruent (red) conditions. Results from a Wilcoxon rank-sum test on the response accuracies suggest that children performed better in the congruent than the incongruent condition (*p* = 0.04, unadjusted). RT results are shown in Figures 6B–D. Figure 6B shows the grand mean RTs in individuals. Figure 6C shows the normalized Cong^4^Cong^5^ RTs and InCg^4^Cong^5^ RTs, with greater RTs following ‘NoGo’ events (i.e., InCg^4^Cong^5^) versus ‘Go’ events (Cong^4^Cong^5^) suggesting the effect of IC. However, no significant differences were found between the grand mean RTs in two conditions, neither was there any significant difference between the group mean InCg^4^Cong^5^ and Cong^4^Cong^5^ RTs. Figure 6D plots (InCg^4^Cong^5^ – Cong^4^Cong^5^) RT in individuals. About half of the children showed greater RT following ‘NoGo’ events, with (InCg^4^Cong^5^ – Cong^4^Cong^5^) RT above the horizontal dash line, and half showed greater RT following ‘Go’ events. The (InCg^4^Cong^5^ – Cong^4^Cong^5^) RT were significantly and negatively correlated with the ages of children. That is, the effect of IC on the RTs of older children was smaller compared to younger children.

### Functional Near-Infrared Spectroscopy Results

#### Functional Near-Infrared Spectroscopy Data Quality

**FIGURE 7:**
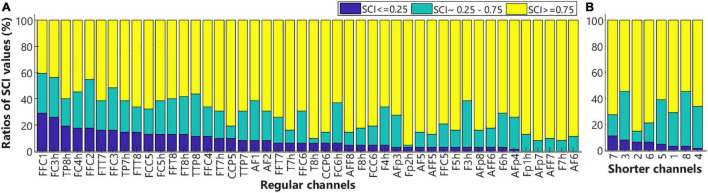
The ratios of scalp coupling index (SCI) values. Bar plots show the ratio of SCI values below 0.25 (blue), within 0.25–0.75 (teal), and above 0.75 (yellow) for the regular **(A)** and shorter **(B)** channels. Low SCI values indicate poor signal qualities.

The quality of signals in each channel was examined by calculating the scalp coupling index (SCI) values that were based on the measures of heartbeat signals from near-infrared light of two different wavelengths in each channel. As heartbeats were the most salient physiological signals in the fNIRS measures, channels that failed to record good heartbeat signals were unlikely to record the neuronal signal of good quality, hence should be excluded from further analyses (Pollonini et al., 2014). Whereas greater SCI values would indicate better signal-to-noise ratios. Figure 7 shows the SCI results in the regular channels (Figure 7A, *n* = 46) and shorter channels (Figure 7B, *n* = 8). For each channel, the ratio of SCI values below 0.25 (poor), between 0.25 and 0.75 (medium), and above 0.75 (good) across all subjects (*n* = 31), were shown in blue, teal, and yellow, respectively. As shown in Figure 7A, SCI values in channels FFC1 and FC3h in close to 30% of subjects were smaller than 0.25. Hence, these two channels were excluded from further analyses for all children. The rest of the regular and shorter channels in most of the subjects showed medium or good SCI values and were included for further analysis.

#### Functional Near-Infrared Spectroscopy Amplitudes

**FIGURE 8:**
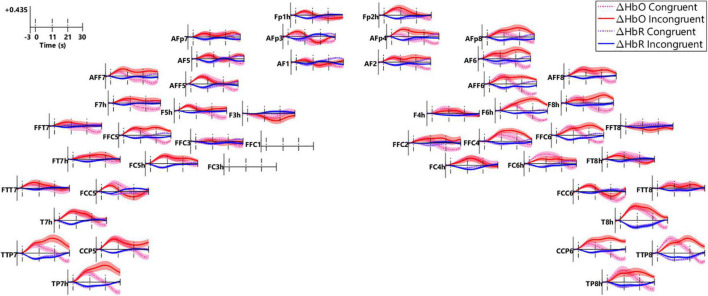
Block-averaged fNIRS responses in all the channels. The small panel plots responses in individual channels (*n* = 46), the locations of which correspond to those in Figure 2B. Block-averaged ΔHbO (red) and ΔHbR (blue) responses in the congruent (dash lines) and incongruent conditions (solid lines) were plotted. Responses were examined in three periods, i.e., the pre-incongruence period (0–11 s, first two vertical dash lines), the intra-incongruence period (11–21 s, the second and third dash lines), and the post-incongruence period (21–30 s, after the third vertical dash line).

Figure 8 plots the block-averaged ΔHbO (red) and ΔHbR (blue) in the congruent (dot lines) and incongruent (solid lines) conditions in the regular channels (*n* = 46), with data from the two channels that showed poor quality (FFC1 and FC3h, Figure 7) excluded. After stimulus onset (first dash line), ΔHbO responses increased and ΔHbR responses decreased in most of the channels and both went back to baseline after speech offset (third dash line), representing a prototype that is consistent with neuronal activity-related changes in hemoglobin. Further, ΔHbO responses in a lot of channels in the right frontal (AFp4, AF2, AFp8, AF6, AFF6, F6h, FFC4, AFF8, F8h, FFC6), a few channels in the left IFG (AFF7, F7h, FFC5, F5h), and in the bilateral auditory areas (TTP7/8, CCP5/6, TP7/8h, T8h) were greater in the incongruent versus congruent conditions in the intra-incongruence and post-incongruence periods. Greater responses in these channels were likely due to the interferences in the ‘NoGo’ events.

**FIGURE 9:**
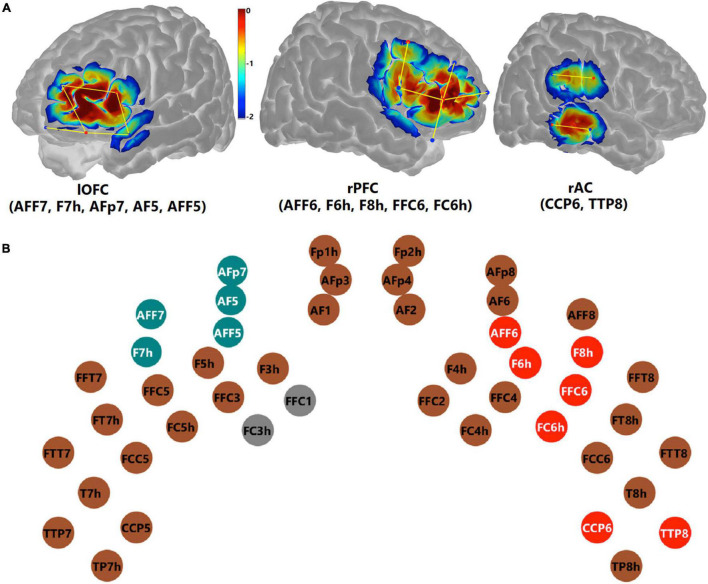
Functional near-infrared spectroscopy measures that revealed inhibitory control. Panel **(A)** shows the channels comprising three ROIs, on the left orbitofrontal cortex (lOFC), the right prefrontal cortex (rPFC), and the right auditory cortex (rAC). The colors are the sensitivity profiles, in log10 mm^– 1^ units, generated from AtlasViewer (Aasted et al., 2015). Panel **(B)** shows montage and marks the channels (dots) which showed a significant effect of inhibitory control. ΔHbO amplitudes in the intra-incongruence period in the teal channels (lOFC) showed a significantly age-dependent effect of inhibitory control. Channels in red showed a significant effect of inhibitory control in the post-incongruent period. Channels in brown showed no significant effect. Channels in gray (FFC1 and FC3h) were excluded from further *analyses* due to poor data quality.

To examine the effect of conflict processing, the maximum (InCg – Cong) ΔHbO amplitude within the post-incongruence period (21–30 s after stimulus onset) was compared with that in the pre-incongruence period (0–11 s) for each channel. Channel-by-channel Wilcoxon sign-rank tests were conducted with the Holm–Bonferroni method being used for multiple comparison correction (*n* = 44). Data from two channels (FFC1 and FC3h, gray in Figure 9B) were excluded due to poor quality (Figure 7). Figure 9B shows individual channels (red) where significantly greater (InCg – Cong) ΔHbO amplitudes were found in the post-incongruence versus pre-incongruence periods (*p*_adjusted_ < 0.05), suggesting the effect of interferences. These channels clustered into two ROIs on the right hemisphere that corresponded to the right PFC (rPFC) that centered at the IFG and DLPFC, and right AC (rAC, Figure 9A).

**FIGURE 10:**
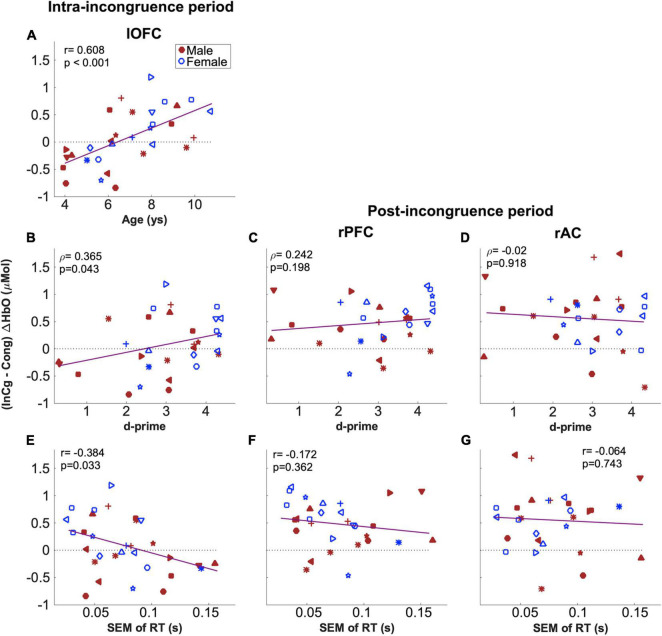
Correlations between (InCg – Cong) ΔHbO amplitudes and the ages and behavioral measures of children. Panels **(A,B,E)** shows the results for (InCg – Cong) ΔHbO amplitudes in the intra-incongruence period in the lOFC with the ages, d-prime values and SEM of RTs for children, respectively. Panels **(C,D)** show the correlation results for (InCg – Cong) ΔHbO amplitudes in the post-incongruence period for the rPFC and rAC with d-prime values. Panels **(F,G)** show the correlational results with SEM of RTs. The same symbols were used to indicate individuals as in previous figures; again, blue is for females and red for males.

The relations between the two behavioral measures from the fNIRS session which were correlated with NIH assessments (i.e., d-prime values and SEM of RTs in Figure 5) and fNIRS measures in the two ROIs which showed a significant effect of inhibitory control (i.e., rPFC and rAC, Figure 9B) were examined. For the fNIRS measures, the mean of the post-incongruence (InCg – Cong) ΔHbO amplitudes across all channels within each ROI was calculated for individuals. As shown in Figure 10, neither of two behavioral measures were correlated with the post-incongruence (InCg – Cong) ΔHbO amplitudes, either in the rPFC (Figure 10B) or in the rAC (Figure 10C). Further, no significant differences in the post-incongruence (InCg – Cong) ΔHbO amplitudes were found between females and males (*p* > 0.05) in either ROI.

To investigate whether older children would recognize the interferences earlier, channel-by-channel analysis was conducted on the (InCg – Cong) ΔHbO amplitudes in the intra-incongruence period. The amplitudes in five adjacent channels (teal, Figure 9B), which were located at the left orbitofrontal cortex (lOFC, Figure 1), showed significant correlations with the ages of children, with *p*_unadjusted_ < 0.05. The means of the intra-incongruence (InCg – Cong) ΔHbO amplitudes across all the channels in the lOFC were calculated. Results found a significant Pearson correlation between the ages of children and the intra-incongruence (InCg – Cong) ΔHbO amplitudes in the lOFC (*r* = 0.608, *p*_adjusted_ < 0.001). This result suggests that children of older ages had greater differences in the ΔHbO amplitudes between the incongruent and congruent conditions compared to children of younger ages. The intra-incongruence (InCg – Cong) ΔHbO amplitudes in the lOFC were also correlated with the d-prime values (*r* = 0.365, *p*_unadjusted_ = 0.043), and with the SEM of RTs (*r* = −0.384, *p*_unadjusted_ = 0.033), though the *p*-values were not significant after multiple comparison corrections.

## Discussion

Limited research links the development of IC in preschool aged children to neuroimaging measures of child brain development. This work builds on an existing literature of IC in children by proposing the use of a multimodal Go/NoGo task using fNIRS, a non-invasive and child-friendly neuroimaging technique.

### Behavioral Measures From the Go/NoGo Functional Near-Infrared Spectroscopy Task Accurately Reflects Inhibitory Control

Children took part in several tests reflecting IC: three standardized, well-known tasks from the NIH Toolbox, and our newly developed multimodal Go/NoGo task while fNIRS data were collected. Significant positive relations between d-prime levels from our Go/NoGo fNIRS task and uncorrected scores in the NIH Flanker inhibitory tests suggests that children who performed well on both tasks have better IC. Here, d-prime values were derived from events #4 and #6, which were ‘Go’ and ‘NoGo’ events in the congruent and incongruent blocks, respectively. Larger d-prime values indicate a better sensitivity in distinguishing ‘NoGo’ from ‘Go’ events immediately after ‘Go’ events, i.e., better *interference suppression* ability and cognitive flexibility (Figure 3). A similar pattern was indicated between d-prime values and uncorrected scores from the Dimensional Card Sort, but this relation did not reach statistical significance. Such differential findings may be due to a lack of power to detect significant relations in our relatively small sample. With a total of 31 children between 4 and 10.8 years of age, and the nature of larger variance across children, our data might be underpowered.

Another measure of enhanced EF might be reflected by the SEMs of RTs. Variability in the RTs has been widely used to reveal attention deficits, with increased variance in RTs being an etiologically important character of children and adolescents with ADHD (see meta-analyses by Kofler et al., 2013). Smaller SEMs (or variances) of RTs indicate more stable responses, likely better sustained attention and focus on the task, hence better IC performance. In the current study, the SEM of RTs were calculated for the ‘Go’ events when children responded correctly and within a certain window after stimulus onset. The SEMs of RTs were related to all three standardized EF tasks; in other words, children who performed better on IC tasks, had more cognitive flexibility, and stronger working memory as assessed using the NIH Toolbox also had more stable responses during the fNIRS Go/NoGo task.

The difference in the mean RTs for ‘Go’ events (event #5) following ‘NoGo’ and ‘Go’ events (event #4), i.e., (InCg^4^Cong^5^ – Cong^4^Cong^5^) RT is another measure to examine the effect of IC (Pires et al., 2018), with increased RTs following ‘NoGo’ versus ‘Go’ events reflecting the effect of interference. Surprisingly, in this group of children no significant differences were found between InCg^4^Cong^5^ RTs and Cong^4^Cong^5^ RTs as measured with the Go/NoGo task (Figure 6C). However, difference in the RTs between following ‘NoGo’ and ‘Go’ events was associated with child age, with larger differences in the younger children (below 6 years of age) compared to older children in this group (Figure 6D). The large variance in children at a young age had poorer attention control, more easily affected by interference, and were not as successful at inhibiting their prepotent responses also with larger variances in RTs. Whereas the lack of interference on the mean RTs observed in the older children may be because the task was relatively easy for them, also reflected by the ceiling response accuracy and d-prime values (Figure 5). As the IC in children develops rapidly between 3 and 6 years of age (see reviews by Garon et al., 2008; Best and Miller, 2010), older children in the current study were perfectly capable of performing the task, but the low task demands might not be able to engage them throughout the task. Future studies assessing the effect of IC based on RT measures should target a narrower age-range as 4–10 years of age is quite broad. Children in this age range, especially at the two ends, may have very different levels of executive functions and attention, a mixed of which could contribute to the non-significant (InCg^4^Cong^5^ – Cong^4^Cong^5^) RT results. Alternatively, the SEM of RTs that is related to the sustained attention throughout the task consisting of six events in a row, might be a better assessment of IC in the current study compared to (InCg^4^Cong^5^ – Cong^4^Cong^5^) RT, which were related to one event per block. A study with more trials and post-incongruence events would be ideal, though hard for young kids to perform. Nonetheless, the significant correlations between the d-prime and SEM of RTs behavioral from the designed Go/NoGo task and the standardized assessments from NIH Toolbox cognition battery suggest that the designed task can successfully reveal EF especially IC in children.

### Functional Near-Infrared Spectroscopy Measures Reveal Links Between the Prefrontal Cortex and Inhibitory Control

Because the Go/NoGo task that we developed accurately reflected EF in children, the next step was to use the fNIRS imaging results to identify regions in the brain associated with EF in children while performing this task. Results comparing fNIRS responses (ΔHbO amplitudes) between the incongruent and congruent conditions indicate an effect of IC in the right PFC (rPFC), covering the right DLPFC and IFG (Figure 9), which are consistent with the function of the right DLPFC for top-down modulation and control implementation (Vanderhasselt et al., 2009) and the right IFG for inhibiting motor responses (Dillon and Pizzagalli, 2007). Neural indicators of IC were evident in a large area and on the right hemisphere for ΔHbO responses, in line with the functions of rPFC. This location is slightly different from previous work in children 3–5 years of age. Fishburn et al. (2019) used a Go/NoGo task and showed IC on both hemispheres, and Li et al. (2017) used a Pet Stroop task and found a significant effect of IC in one channel on the left DLPFC. As summarized earlier, the left DLPFC may be associated with the expectation and preparation for upcoming changes in the attentional set (see reviews by Vanderhasselt et al., 2009), i.e., conflict monitoring. The Li et al. (2017) results showed significant results in the left DLPFC, but the current study did not potentially for several reasons. First, while studies implemented the idea of incongruent animal images and sound, Li et al. (2017) used a forced-choice task with four options (four animals), which is more complicated compared to our Go/NoGo task. The complexity of a task, especially for children 3 years of age who may have poor IC, may contribute to the limited significance found in Li et al. (2017). Second, previous studies had different ratios of congruent versus incongruent trials, with a ratio of 1:1 in Li et al. (2017) and 2.5:1 in the current study. More frequent congruent trials in the current study, as recommended when examining IC (Bugg et al., 2008), could have increased the sensitivity to goal maintenance in the congruent condition, hence build up stronger contrast of conflicts when the forthcoming events are unanticipated. Third, our designed Go/NoGo task included extra interference from an informative spatial cue that correctly or incorrectly predicted the location of forthcoming images and sounds, i.e., interference between spatial and audio-visual cues of locations, which was not available in Li et al. (2017). The processing of conflict cues from multiple aspects could recruit greater activity in the PFC, which indicated some level of age-independence (Figure 10B). Overall, our findings suggest that our newly designed Go/NoGo task can robustly reveal neural correlates of IC in the rPFC in children between 4 and 10 years of age.

We have to admit that there were potentialities that the reported brain regions and channels in the current study might not correspond to the same cortical regions on each child. An optimal way would be to mark the channel and optode locations for each child during fNIRS data collection, which would then be co-registered to his/her owe structural MRI. However, we did not use a spatial digitizer for every child because the long time for co-registration may have led to the loss of young children’s attention, and the entire study length with NIH assessments, practice for fNIRS and real tasks, were already long. To reduce potential errors, first, we posited our optodes on NIRScaps based on 10–10 system (Figure 1A). We then registered the channel locations to the head using AltasViewer software, which provided information about channel coverage. During data collection, we used NIRScaps based on children’s head circumferences and made sure each cap was a snug fit. We also centralized the cap with reference to Nz, Iz, Cz, and LPR and RPR. We hope these steps could improve the accuracy of the brain regions where our data were recorded from.

### Our Task Is Not Inhibitory Control Specific

We further examined the relations between fNIRS and behavioral measures of IC in this group of children, but found that neither of the two behavioral measures were correlated with the post-incongruence (InCg – Cong) ΔHbO amplitudes in the rPFC or rAC (Figures 10C,D,F,G). These results suggest that, activity in the rPFC was indicative of the processing of prepotent responses, but independent of behavioral performance. We posit that fNIRS measures may be neural markers that reveal general cognitive procedures including but not limited to IC. Hence fNIRS measures in these two regions were not correlated with the behavioral measures from the same task. For instance, it can be challenging to disentangle cognitive flexibility from IC, especially in young children. According to Miyake et al. (2000), the three core components of EF – cognitive flexibility, working memory, and IC may be separable constructs but serve related functions. Consistent with this theory, children and adolescents between 8 and 13 years of age exhibit EF behaviors that support a unity-and-diversity model (Lehto et al., 2003). Whereas another study found that in children 7–15 years old, IC did not load any additional factor beyond a model of EF based on working memory and cognitive flexibility (Huizinga et al., 2006). It is possible that the common factor underlying EF components reflects individual differences in maintaining and managing goals, which are particularly important for IC, thus IC did not load an additional factor (Friedman and Miyake, 2017; McKenna et al., 2017). Alternatively, the tests that were used to tap IC also involved working memory and cognitive flexibility, depending on the complexity of the tests (Garon et al., 2008). For instance, the traditional Go/NoGo response task taps *response* inhibition (Figure 3), while the relatively more complicated Stroop task taps both *interference suppression* and *cognitive flexibilities*, i.e., switching between different dimensions and modalities. The Pet image/sound Stroop task in study by Li et al. (2017), which required children to categorize animals based on their sounds rather than the prepotent images, was claimed to target cognitive flexibility. However, if we consider the definition of IC *as* the ability to stop impulses and prepotent responses – *response inhibition* – *and* the ability to resist interference from misleading or irrelevant information – *interference suppression*, we then argue the task in Li et al. (2017) also targeted IC. In the current study, we propose that our designed Stroop and Go/NoGo was meant to mainly target IC, as well (Figure 3).

We, however, considered the effect of surprisal reaction to the incongruent trials on the cortical activity. The designed Go/NoGo task was configured to involve congruent cues from multiple dimensions, with frequent trials (more frequent than trials with interferences), and informative cues could quickly build up anticipations of the forthcoming events, which in turn could improve response efficiency. However, when the forthcoming events were unanticipated and ‘violated’ the expected sequence of events, children could be surprised (Itti and Baldi, 2009). Anticipation is associated with the cortico-striatal-thalamic network, and surprises have activated greater activity in the PFC and parietal regions, which overlap with the network activated by conflicts (Fan et al., 2007). In the auditory field, infrequent deviants in homogenous sequences of sounds could activate a mismatch field covering the temporal and parietal lobes, with greater activity in the right versus left hemisphere (Levänen et al., 1996; Parmentier, 2014). The auditory processing of surprise could affect cortical activity in the PFC for inhibitory control. However, results in Fan et al. (2007) suggest that anticipation modulates overall activity in the executive control network but does not interact with conflict processing. Therefore, the significant results that we found in the rPFC are probably due to IC in the incongruent condition rather than a surprisal reaction.

### Age and Sex Differences in Inhibitory Control

That older children in our study had smaller variance in RT (hence, better attentional and inhibitory control) suggests that older children with more developed EF might be more alert to and prepared for potentially unexpected forthcoming events (MacDonald et al., 2000). The next step, then was to examine whether this relation continued in the neural processing of information. As might be expected, our fNIRS results demonstrated greater intra-incongruence (InCg – Cong) ΔHbO amplitudes in the left OFC (lOFC, Figure 9) as the ages of children increased. This suggests that older children show greater responses and better IC in the PFC compared to younger children. Similar to our results, an fMRI study also found a significant and age-dependent effect of IC in the left lateral PFC (Adleman et al., 2002). In their study, Adleman et al. (2002) used the color/word Stroop task and showed significant and positive correlations between the ages in children and young adults between 7 and 22 years of age and IC in the left lateral PFC and ACC. Together, these results suggest that the development of IC for prepotent responses is related to the maturation of the left lateral PFC. Our results also indicated significant correlations between the intra-incongruence (InCg – Cong) ΔHbO amplitudes in the lOFC and two behavioral measures recorded from the fNIRS session (i.e., d-prime values and SEM of RTs *p*-values unadjusted). These results and results above suggest that, cortical activity related to IC in the lOFC is age, and hence, performance dependent; whereas activity in the rPFC is independent of age and behavioral performance.

Finally, the development of EF for children at this age generally favors girls at an earlier age (Else-Quest et al., 2006). In this study, girls showed trends of better performance in the Flanker inhibitory control task, greater d-prime values, and smaller variances in RTs in the Go/NoGo task compared to boys (Figure 4). However, no significant differences were found between the two sexes after multiple comparison corrections. The lack of significance could be due to the small sample sizes (14 females, 17 males) across a large age range in the current study. Moreover, though differences have been observed from behavioral measures of EF in previous studies, the effect sizes are small to moderate (Else-Quest et al., 2006); hence we may not be sensitive to such differences with a relatively small group across a large age range (4–10.8 years). In addition, sex differences might affect some aspects of EF for instance processing speed and arousal but not inhibitory control or working memory (Brocki and Bohlin, 2004), however, no significant differences were observed in the current study.

## Conclusion

This study developed a multimodal Go/NoGo task to examine inhibitory control (IC) in children 4–10 years of age using fNIRS. Our study for the first time compared behavioral measures recorded in the designed task with standardized assessments from NIH Toolbox cognition battery and showed significant correlations between them. fNIRS measures revealed a significant but age-independent effect of IC in the rPFC, and an age-dependent effect of IC in the lOFC, consistent with findings from previous neuroimaging studies. We verified that the task we designed is suitable to reveal IC processes in children of this age range and that fNIRS is a promising tool to examine the development of executive functions. Our results help to fill the gap of neuroimaging measures of EF in young children who are hard to test using other techniques.

## Data Availability Statement

The raw data supporting the conclusions of this article will be made available by the authors, without undue reservation.

## Ethics Statement

All experimental protocols were within standards set by the National Institutes of Health and approved by the University of Wisconsin–Madison’s Human Subjects Institutional Review board. Written informed consent to participate in this study was provided by the participants’ legal guardian/next of kin.

## Author Contributions

XZ, DD, and EP conceived the study. XZ, LH, CP, and MD collected all the data. XZ analyzed the data. AA and RL contributed to the discussions of the project. All authors contributed in the writing and editing of the manuscript. All authors contributed to the article and approved the submitted version.

## Conflict of Interest

The authors declare that the research was conducted in the absence of any commercial or financial relationships that could be construed as a potential conflict of interest.

## Publisher’s Note

All claims expressed in this article are solely those of the authors and do not necessarily represent those of their affiliated organizations, or those of the publisher, the editors and the reviewers. Any product that may be evaluated in this article, or claim that may be made by its manufacturer, is not guaranteed or endorsed by the publisher.
